# Practice Pattern Variability in the Use of Pulmonary Arterial Catheters in Cardiac Surgery

**DOI:** 10.1053/j.jvca.2025.08.013

**Published:** 2025-08-13

**Authors:** Emily J. MacKay, Bo Zhang, Joseph M. Beaty, Katelyn A. Devine, Vikas O’Reilly-Shah, Michael R. Mathis, Wilson Y. Szeto, Peter W. Groeneveld, John G. Augoustides

**Affiliations:** * Department of Anesthesiology and Critical Care, Perelman School of Medicine at the University of Pennsylvania, Philadelphia, PA; † Vaccine and Infectious Disease Division, Fred Hutchinson Cancer Center, Seattle, WA; ‡ Perelman School of Medicine, University of Pennsylvania, Philadelphia, PA; § Department of Anesthesia and Critical Care, University of Chicago, Chicago, IL; ‖ Department of Anesthesiology and Pain Medicine, University Washington, Seattle, WA; ¶ Department of Anesthesiology, University of Michigan, Ann Arbor, MI; ** Division of Cardiovascular Surgery, Perelman School of Medicine at the University of Pennsylvania, Philadelphia, PA; †† Division of General Internal Medicine, Perelman School of Medicine at the University of Pennsylvania, Philadelphia, PA; ‡‡ Penn’s Cardiovascular Outcomes, Quality and Evaluative Research Center, University of Pennsylvania, Philadelphia, PA; §§ Leonard Davis Institute of Health Economics, University of Pennsylvania, Philadelphia, PA; ‖‖ Corporal Michael J. Crescenz Veterans Affairs Medical Center, Philadelphia, PA

**Keywords:** pulmonary arterial catheterization, hemodynamic monitoring, cardiac surgery

## Abstract

**Objectives:**

To quantify intraoperative pulmonary arterial catheter (PAC) use during cardiac surgery and identify hospital-, anesthesiologist-, and patient-level factors associated with PAC utilization.

**Design:**

A cross-sectional, observational study using generalized logistic mixed models to examine variations in PAC use.

**Setting:**

Fifty-three US academic hospitals participating in the Multicenter Perioperative Outcomes Group (MPOG) national registry

**Participants:**

145,343 adult patients undergoing cardiac surgery between January 1, 2016, and December 31, 2022.

**Intervention(s):**

Receipt of intraoperative PAC, defined by ≥60 minutes of physiologically plausible pulmonary arterial pressures.

**Measurements & Main Results:**

The primary outcome was PAC utilization. Mixed-effects logistic regression quantified fixed-effect predictors, and variation attributable to anesthesiologists and then to anesthesiologists nested within a hospital was characterized using median odds ratio (MOR). Of the 145,343 cardiac surgeries performed across 53 hospitals, 104,626 (72%) included PAC monitoring. PAC use varied widely across hospitals (0–98%) and across anesthesiologists (0–100%). PAC was used most frequently in heart transplants (94%) and lung transplants (87%) and least frequently in pulmonic valve procedures (30%). A patient’s likelihood of receiving a PAC was influenced most strongly by hospital (MOR, 15.00; 95% confidence interval [CI], 8.98–28.32), with substantially less variation attributable to an anesthesiologist within the same hospital (MOR, 1.70; 95% CI, 1.61–1.81).

**Conclusions:**

Intraoperative PAC monitoring is used in nearly three-quarters of cardiac surgeries at US academic centers, with hospital practice pattern the factor most closely associated with PAC utilization.

PULMONARY ARTERIAL CATHETERIZATION (PAC) is an invasive hemodynamic monitoring technique that allows real-time assessment of cardiovascular physiology by directly measuring pulmonary artery pressures, pulmonary capillary wedge pressure, and mixed venous saturation and calculating cardiac output.^1,2^ These parameters offer a comprehensive assessment of right and left heart function, pulmonary vascular resistance, and systemic hemodynamics, making PAC a potentially valuable tool in the perioperative management of high-risk surgical patients, particularly those undergoing complex cardiac procedures.^3^

Despite these physiologic advantages and long-standing integration into cardiac anesthesia practice, the routine intraoperative use of PAC remains controversial.^4–10^ A substantial body of observational research has yielded conflicting findings regarding the association between PAC monitoring and post-operative clinical outcomes.^4–10^ In noncardiac surgical populations, enthusiasm for PAC use declined following the publication of several randomized controlled trials (RCTs) that consistently failed to demonstrate significant clinical outcome benefits while simultaneously raising concerns over potential complications, including arrhythmias, catheter-related infections, and pulmonary artery rupture.^11–13^ This evidence has led to a substantial reduction in PAC use across noncardiac surgical and intensive care settings.^14^

In contrast, PAC use in cardiac surgery historically has remained high. Older observational studies published before 2015 reported intraoperative PAC utilization rates ranging from 50% to 75%, despite similar ambiguity around clinical benefit in this population.^6,7,15^ Recent data on national PAC utilization are sparse, however. To date, only a single 2023 national analysis using administrative claims reported a markedly lower PAC utilization rate of 7% in cardiac surgery patients.^16^ These discrepancies reflect both changes in practice and the limitations of available data sources and highlight the need for a comprehensive, contemporary understanding of PAC utilization in cardiac surgery.

Accordingly, the objectives of this study were to (1) determine the current rate of intraoperative PAC use across a nationally representative sample of US academic cardiac surgical centers participating in the Multicenter Perioperative Outcomes Group (MPOG) registry and (2) identify patient-, hospital-, and anesthesiologist-level factors associated with PAC utilization during cardiac surgery.

## Methods

This study was reviewed and approved by both the University of Pennsylvania and University of Washington Institutional Review Boards. Informed consent was waived given the deidentified nature of the data. The study was designed and reported in accordance with the Strengthening the Reporting of Observational Studies in Epidemiology (STROBE) guidelines.^15^

### Data Source

Data were queried from the national MPOG data registry, which contains more than 20 million anesthesia records from a diverse sample of US hospitals.^16^ Participating hospitals contribute data to MPOG using automated data extraction methods from the local electronic medical record (EMR). Quarterly data audits and quality checks are done by MPOG to ensure the accuracy of patient demographics, preexisting comorbidities, surgical procedures, and intraoperative data fields (procedures, hemodynamic data, medications, fluids, etc) and clinical outcomes.^17^

### Study Cohort

The study cohort included adult patients aged 18 years or older who underwent cardiac surgery between January 1, 2016, and January 1, 2023. Inclusion was restricted to data that met the MPOG perioperative research standard for data fidelity at the time of data query^18^ and to cases classified as having undergone “open cardiac surgery.”^19^ Cases were excluded if any of the following criteria were met: (1) missing starting anesthesia attending; (2) missing case status (emergent, urgent, elective); (3) underwent surgery at a hospital that performed <25 surgeries annually; (4) cardiopulmonary bypass (CPB) duration <20 minutes (if CPB was used); (5) chest exploration or chest closure case; and (6) arrived while intubated. These exclusions were applied to focus the analysis on routine cardiac surgeries in which PAC placement decisions are most discretionary. Specifically, cases with very short CPB duration were excluded to eliminate atypical procedures, patients arriving intubated were excluded to avoid mis-attributing preexisting PACs to intraoperative placement, and hospitals with extremely low surgical volume were excluded to reduce noise from outlier practice environments.

### Outcome Variable

The study’s primary outcome was receipt of a PAC, defined by ≥60 minutes of physiologically plausible pulmonary artery systolic (20–100 mmHg), diastolic (0–60 mmHg), or mean (10–80 mmHg) pressure. This outcome was validated by manual chart review by 2 independent reviewers from 2 different institutions, the Hospital of the University of Pennsylvania and the University of Washington Medical Center, to ensure PAC data fidelity.

### Predictor Variables

Fixed-effects variables encompassed both patient and hospital factors. Patient-level covariates included (1) demographics (eg, age, sex, body mass index, American Society of Anesthesiology physical status); (2) surgical procedure(s) (eg, valve [aortic, mitral, tricuspid, pulmonic] repair or replacement, coronary artery bypass graft (CABG) surgery, heart transplant, lung transplant, proximal aortic surgery, descending thoracic aortic surgery, whether or not the case was a redo sternotomy, emergency, or done on a weekend versus a weekday); (3) comorbidities most relevant to cardiac surgical patients (eg, cardiac arrhythmias, chronic pulmonary disease, congestive heart failure, pulmonary circulatory disorders, valvular disease, cerebrovascular disease, coronary arterial disease); and (4) preoperative laboratory test values (eg, albumin, blood urea nitrogen, glucose, potassium, hemoglobin, hemoglobin A1c, international normalized ratio, platelet count). Hospital-level fixed-effects covariates included surgical volume and staffing by a certified registered nurse anesthetist versus a resident trainee. For the generalized logistic mixed model (GLMM) analyses, random effects were incorporated by allowing each hospital and the anesthesiologist who started the case to be incorporated as random intercepts.

### Data Validation

In addition to standard MPOG methods used to validate covariates,^17^ the PAC variable underwent additional validation. To accomplish this rigorously, 2 random subsets were derived from the larger dataset to manually check against local intraoperative records. The 2 subsets of data were from the Hospital of the University of Pennsylvania and the University of Washington Medical Center. By manual EMR review, the reference standard definition for PAC by manual chart review was confirmed if the chart had a procedural note for placement of a PAC and at least 1 of the following: (1) recorded SvO_2_ value and (2) physiologic systolic, diastolic, or mean pulmonary arterial pressure(s) at a minimum of 3 timepoints during surgery: 10 minutes after PAC placement (or 10 minutes prior to surgical incision), 30 minutes prior to CPB (or mid-case if CPB was not used), and 30 minutes after CPB (or 30 minutes before procedural end time if CPB was not used). Manual chart review was performed by 2 investigators, 1 each from the Hospital of the University of Pennsylvania (J.M.B.) and the University of Washington Medical Center (K.A.D.). From the sample of chart reviews, 2 × 2 contingency tables were con structed to compare the MPOG-defined PAC variable with the manual EMR-defined PAC reference standard. Performance characteristics for the identification of intraoperative PAC were calculated; these statistics included sensitivity, specificity, positive predictive value, and negative predictive value, along with exact, binomial 95% confidence interval (CI).

### Statistical Analyses

The proportion of patients who underwent cardiac surgery with PAC versus without PAC was calculated. Baseline patient and hospital characteristics were compared between the 2 groups using descriptive statistics. For continuous variables, single imputation with mean was used for fixed-effects variables with <5% missingness,^20^ and multiple imputation with Python autoimpute was used for variables with 6% to 15% missingness.^21,22^

To evaluate the association between intraoperative PAC use and both patient-level and anesthesiologist/hospital-level characteristics, GLMM was used at 2 hierarchical levels. The primary analysis was a 2-level GLMM that included fixed effects for patient-level and selected hospital-level covariates and accounted for clustering of patients by the anesthesiologist who started the case. The secondary analysis used a 3-level GLMM that incorporated random effects for anesthesiologists nested within hospitals. This model also included fixed effects for patient- and hospital-level covariates, allowing quantification of the variation attributable to both anesthesiologists and institutions. The effects of individual patient-level covariates were estimated using the adjusted odds ratio (OR), which can be interpreted in the standard manner. The influence of anesthesiologist-level and hospital-level clustering was quantified using the median odds ratio (MOR), which is directly comparable to the ORs of fixed-effects variables.^23,24^ Used in previous research to study the impact of provider practice patterns on an exposure of interest,^25–28^ the MOR quantifies the extent of variability in intraoperative PAC use attributable to different levels of clustering. In the 2-level model, which included anesthesiologist as a random effect, the MOR reflects variation in PAC use between individual anesthesiologists across all hospitals. In the 3-level model, 2 random effects were included: anesthesiologists nested within hospitals and hospitals themselves. In this model, 2 MORs are reported. The anesthesiologist-level MOR captures variability in PAC use among anesthesiologists within the same hospital, while the hospital-level MOR reflects variability attributable to differences between hospitals—including cases managed by different anesthesiologists.

All hypothesis testing was 2-sided, with significance set at p < 0.05. Data cleaning and processing were conducted in Python version 3.12.2. All statistical analyses were conducted using Stata 18.0 (StataCorp). Statistical analysis was performed from April 2024 to March 2025. A link to the GitHub code repository is provided in Appendix A, Supplementary Material.

## Results

### Study Population

**Fig 1.**
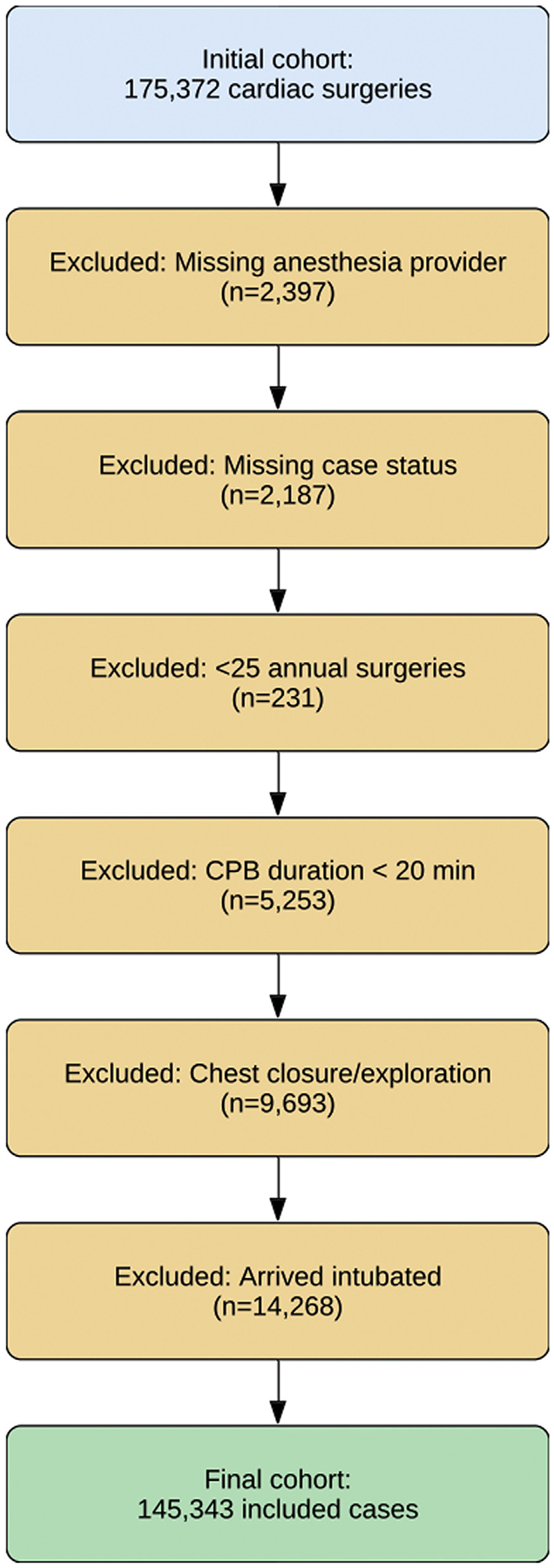
Study flow diagram showing cohort selection, exclusion criteria, and the final analytic sample. CPB, cardiopulmonary bypass.

**Fig 2.**
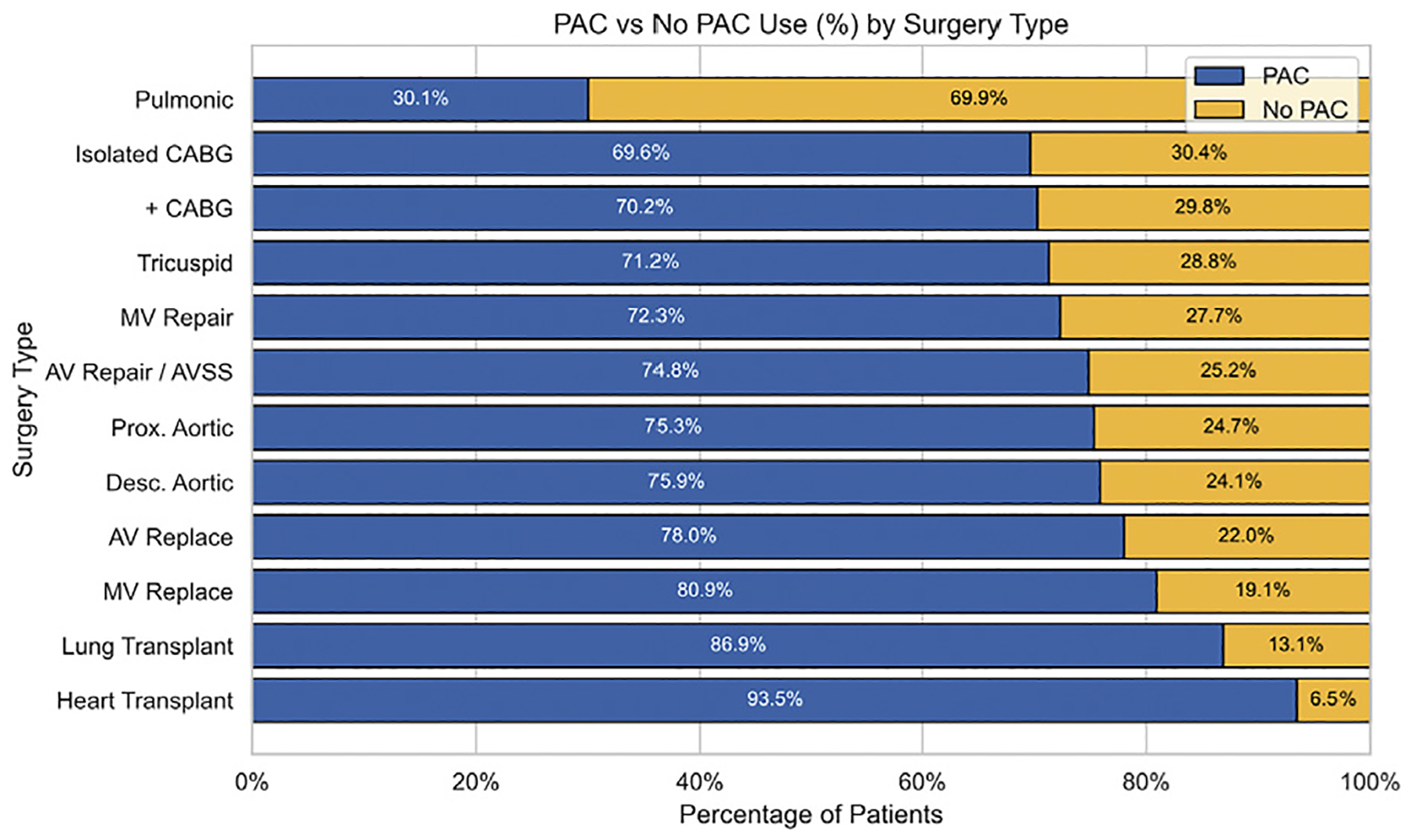
Stacked bar chart illustrating intraoperative pulmonary arterial catheter (PAC) use by surgical procedure. AV, aortic valve; AVSS, aortic valve–sparing surgery; CABG, coronary artery bypass graft; MV, mitral valve.

Following exclusions (Fig 1), the study cohort comprised 145,343 patients who underwent cardiac surgery. Intraoperative PAC monitoring was used in 104,626 patients (72%) and not used in 40,717 patients (28%). The total cohort of 145,343 patients included 99,630 males (68.54%), with a racial composition of 13,213 black (9.10%), 4,778 Asian (3.29%), and 103,112 white (70.95%) patients (Table 1). Compared with patients who did not receive PAC, those who received PAC had higher rates of presurgical comorbidities (eg, arrhythmias, congestive heart failure, pulmonary circulatory disorder, peripheral vascular disease), and were more likely to have undergone surgery at a hospital with a high surgical volume (Table 1). The rate of PAC use varied by surgical type, with the lowest rates observed in patients undergoing pulmonic valve surgery or isolated CABG surgery and the highest rates seen in patients undergoing lung or heart transplantation (Fig 2).

### Variation Among Hospitals and Anesthesiologists

**Fig 3.**
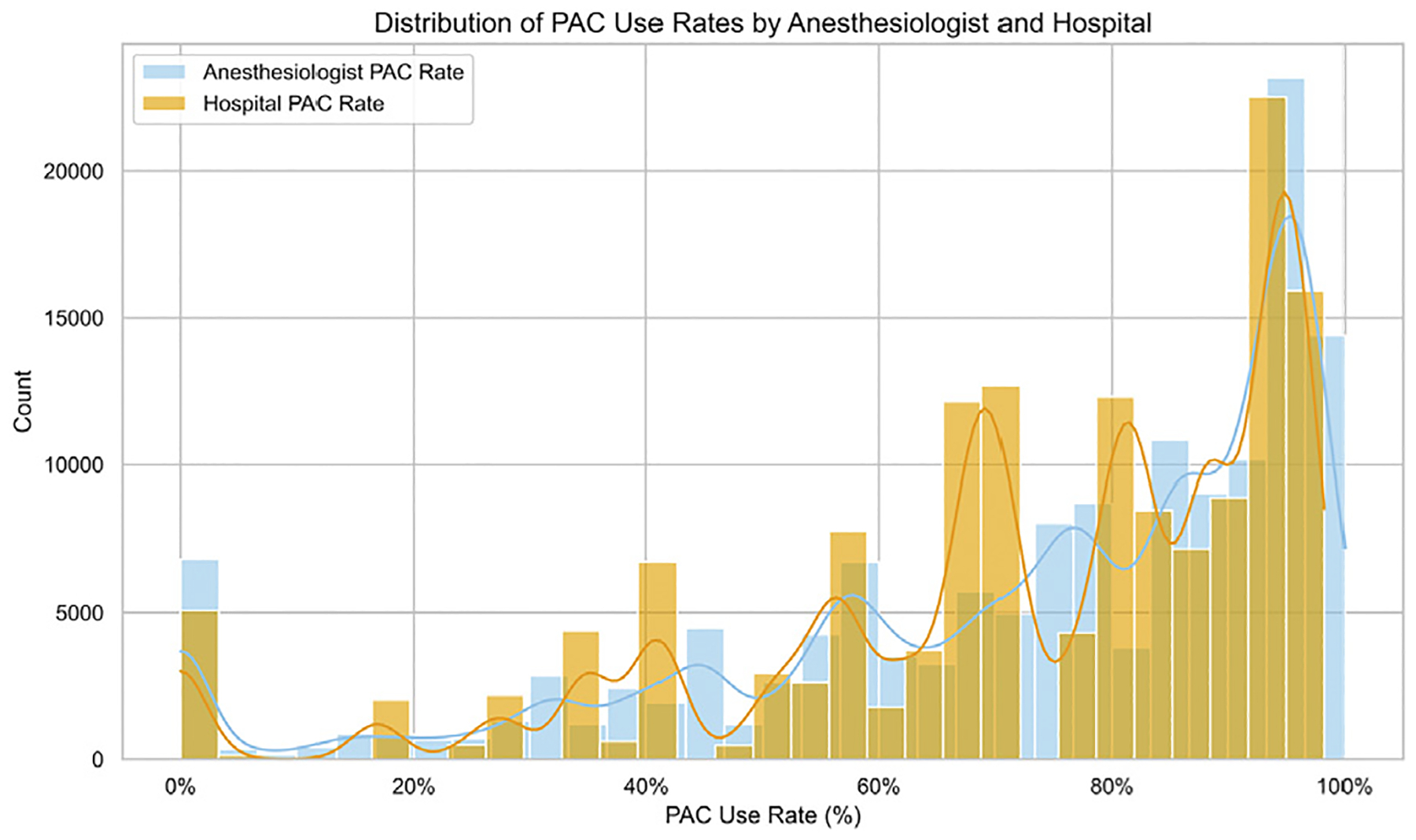
Histograms and density plots illustrating the frequency of pulmonary arterial catheter (PAC) use by hospital (*orange bars and line*) and by individual anesthesiologist (*blue bars and line*).

There was substantial variation among both hospitals and anesthesiologists in the use of intraoperative PAC monitoring. The frequency of intraoperative PAC ranged from 0% to 98% across 53 hospitals (median, 80%; interquartile range [IQR], 61%-92%) and from 0% to 100% across 1,722 individual anesthesiologists (median, 80%; IQR, 58%-94%) (Fig 3).

### Validation of PAC Variable

Data validation was conducted on 411 cases, including 264 from the Hospital of the University of Pennsylvania and 147 from the University of Washington Medical Center, with an approximately equal distribution across all study years. Of the 346 surgeries performed with a PAC, the MPOG-PAC variable defined using the prespecified pulmonary arterial pressure criteria correctly classified 344 cases in which a PAC was used (ie, 344 of 346 true positives) and incorrectly classified 2 cases as having used a PAC when no PAC was used (ie, 2 of 346 false positives). Of the 65 surgeries performed without a PAC, the MPOG-PAC variable correctly classified 53 cases as not having used a PAC (ie, 53 of 65 true negatives) and incorrectly classified 12 cases as having a PAC when no PAC was used (ie, false positive). The study’s pressure-based definition of intraoperative PAC had high sensitivity (99.42%; 95% CI, 98.69%-100.15%), high positive predictive value (96.63%; 95% CI, 94.88%-98.37%), and high negative predictive value (96.36%; 95% CI, 94.55%-98.17%), but lower specificity (81.54%; 95% CI, 77.79%-85.29%).

### Two-Level Predictive Model for Intraoperative PAC During Cardiac Surgery

Among the 145,343 patients undergoing cardiac surgery, the present 2-level (patient- and hospital-level fixed effects plus individual anesthesiologist random effects) analysis identified the individual anesthesiologist as a strong predictor of intraoperative PAC, with an MOR of 7.70 (95% CI, 6.82–8.76) (Table 2). That is, for 2 otherwise identical patients undergoing surgery and cared for by 2 randomly selected anesthesiologists (from any hospital), the odds of PAC differed by more than 7-fold. At the patient level, 2 clinical factors had an even greater influence on PAC use than the anesthesiologist: heart transplantation (MOR, 9.70; 95% CI, 7.12–13.30) and lung transplantation (MOR, 9.58; 95% CI, 7.58–12.15) (Table 2). Full results of the two-level GLMM analysis are provided in the first column of Table 2.

### Three-Level Predictive Model for Intraoperative PAC During Cardiac Surgery

Among the 145,343 patients undergoing cardiac surgery, the study’s 3-level (patient- and hospital-level fixed effects plus anesthesiologist nested within hospital random effects) analysis revealed that the greatest source of PAC variability was between hospitals. In this model, the MOR for PAC use between hospitals (accounting for 2 randomly selected hospitals and their anesthesiologists on staff) was 15.00 (95% CI, 8.98–28.32) (Table 2); that is, there was a 15-fold difference in the odds of receiving a PAC between 2 otherwise identical patients treated at different hospitals. In contrast, the MOR for PAC across anesthesiologists within the same hospital was only 1.70 (95% CI, 1.61–1.81) (Table 2), reflecting a relatively modest variation between anesthesiologists practicing at the same hospital. At the patient level, although no individual factor exceeded the hospital-level variation, heart transplantation (MOR, 10.72; 95% CI, 7.77–14.81) and lung transplantation (MOR, 8.94; 95% CI, 7.12–11.25) were among the strongest predictors of PAC receipt (Table 2). Notably, the reduction in anesthesiologist-level MOR from 7.70 in the 2-level model to 1.70 in the 3-level model suggests that much of the variation previously attributed to anesthesiologists is in fact driven by hospital-level differences. Full results from the 3-level analysis are presented in the second column of Table 2.

## Discussion

In this national population-based cross-sectional study of 145,343 patients undergoing cardiac surgery at 53 primarily academic hospitals participating in the MPOG national database, intraoperative PAC monitoring was used in 72% of the cases. The present multilevel analyses uncovered substantial across-hospital variation in PAC use, with the hospital itself ultimately emerging as the strongest predictor of whether a patient underwent cardiac surgery with PAC versus without PAC. These findings underscore the continued reliance on intraoperative PAC monitoring in the US cardiac surgery patient population and suggest that institutional practice patterns are more influential than surgical complexity or individual anesthesiologist in determining whether PAC is used intraoperatively.

The 72% rate of PAC use seen in this study aligns with earlier observational studies conducted before 2015, which reported PAC use in 50% to 75% of cardiac surgeries.^6,7,29^ However, this finding diverges sharply from more recent multicenter studies, such as the 2023 US national claims-based analyses by Beydoun et al,^30^ which reported a PAC rate of 7.15%, and the 2025 Australian national claims-based analysis by Perry et al,^31^ which reported a PAC rate of 47.4%. These profound discrepancies in PAC use (US, 7%;^30^ Australia, 47%;^31^ 72% in the current study) is likely attributable to differences in data sources, outcome definitions, and a higher proportion of academic centers. Moreover, although to date no study has validated procedural codes for central line or PAC placement explicitly, 1 investigation did observe that while major procedures (eg, surgeries) are well captured, minor procedures (eg, central lines, PACs) are frequently undercoded.^32^ Thus, administrative claims data likely underreport these monitoring line procedures, due to either lack of billing or inconsistent coding, potentially contributing to the markedly lower PAC rates observed in claims-based analyses.^30,31^ In contrast, the present physiologic data-driven definition of PAC use, validated through manual chart review, provides greater sensitivity and specificity compared to claims-based identification of minor line procedures.^32^

Perhaps the most striking finding of the present our study is the magnitude of practice variation in PAC use across hospitals. After adjusting for patient case mix, surgical complexity, and anesthesiologist, there was a 15-fold variation in PAC use between 2 identical patients treated at different hospitals. This degree of variation rivals or exceeds that reported in studies of intraoperative transesophageal echocardiography,^33,34^ benzodiazepine use,^27^ and inotrope administration^28^ in cardiac surgical settings. The finding that anesthesiologist-level variation, although present, was far smaller (MOR, 1.70) further supports the dominant role of institutional norms in shaping PAC use. This pattern suggests that despite decades of debate and investigation, the decision to use intraoperative PAC monitoring remains highly discretionary and is likely influenced by institutional culture, legacy training, and local risk tolerance rather than by strong evidence-based guidelines. The MOR analysis used in this study helps quantify this discretionary space by providing a patient-centered, interpretable metric of variability not captured by conventional fixed-effects modelling.

Although hospital-level factors dominated in predictive power, specific patient and procedural characteristics also were associated with PAC use. Heart and lung transplantation, complex aortic procedures, and higher American Society of Anesthesiology physical status score were all strongly associated with increased odds of PAC monitoring. These associations are clinically intuitive and likely reflect greater hemodynamic risk and the need for close monitoring in these high-acuity settings. Conversely, isolated CABG surgery—a procedure associated with more stable hemodynamics and lower acuity—was more frequently performed without PAC monitoring. These results highlight a partial alignment between monitoring intensity and procedural risk, although the large between-hospital variation suggests that this alignment is far from universal.

The role of PAC monitoring in cardiac surgery remains controversial, owing in part to inconsistent findings across older observational studies and a paucity of randomized controlled trials.^4–10^ A recent systematic review similarly concluded that the evidence for PAC use remains unknown.^10^ In this context, the present findings do not directly address PAC effectiveness but do demonstrate that use is widespread, practice patterns are highly variable, and that utilization is more reflective of where a patient receives care than who they are or how sick they are. These insights reinforce the need for prospective randomized controlled trials or well-designed, matched retrospective comparative effectiveness research to determine which patients, if any, benefit most from PAC use in the modern era.

This study has several strengths. First is its leverage of a large, diverse, and validated multicenter registry with granular intraoperative data, enabling precise identification of PAC use through physiologic criteria. The outcome variable was rigorously validated through manual chart review at 2 independent institutions and demonstrated excellent performance characteristics. Second, the analytic approach used hierarchical modeling with random effects, allowing quantification of both patient- and provider-level contributions to PAC practice pattern variation. Third, the study uniquely quantifies the influence of the individual anesthesiologist on PAC use by using deidentified provider identifiers available in the MPOG database. Such identifiers are generally unavailable in administrative claims data and are entirely absent from the Society of Thoracic Surgeons national registry. While deidentified surgeon identifiers are available in MPOG, prior studies have shown that surgeon intraoperative practices are closely aligned (and highly correlated with) institutional norms.^33,34^ In contrast, anesthesiologist identifiers in MPOG allow for meaningful assessment of provider-level variation using both 2-level and 3-level GLMMs to better isolate the specific contribution of anesthesiologists to PAC use during cardiac surgeries.

Despite these strengths, the present findings should be interpreted considering certain limitations. First, as with all observational studies, residual confounding may persist despite adjustment. Second, the analysis did not include clinical outcomes, and thus whether variation in PAC use translated into differences in patient morbidity or mortality cannot be assessed. Third, although the dataset encompassed a broad sample of US cardiac surgical centers, it may overrepresent academic centers and underrepresent centers with lower surgical volume or private hospitals not participating in MPOG. Fourth, owing to unavailability in the MPOG dataset, the impact of echocardiographic parameters (eg, left ventricular ejection fraction, right ventricular systolic function) on PAC use could not be evaluated. Fifth, because the MPOG database fully deidentifies contributing institutions and does not retain geographic identifiers, the influence of geographic region or regional socioeconomic factors on PAC utilization could not be assessed. As a result, whether differences in PAC use may be partially explained by regional disparities in healthcare access, resource availability, or population-level comorbidity burden remains unclear. Sixth, mechanical circulatory support was not included as a variable in the GLMM analyses because whether this support was planned or unplanned could not be determined, and thus it would not accurately reflect the initial decision to use a PAC at the initiation of the surgical procedure. Seventh, although the physiologic definition of PAC use was validated, it may still misclassify rare edge cases (eg, catheters removed prior to recording sufficient pressures, erroneously labeled monitors).

## Conclusion

This analysis of 145,343 patients undergoing cardiac surgery found that intraoperative PAC monitoring is common in US academic centers, used in nearly three-quarters of cases. Hospital site emerged as the strongest predictor of PAC use, overshadowing individual patient risk factors and even individual anesthesiologist. These results highlight substantial, unexplained variation in clinical practice and suggest that institutional norms continue to exert a powerful influence on intraoperative monitoring decisions. Future research should focus on identifying whether, and in which patients, PAC use improves outcomes, and on generating evidence that can inform more consistent, evidence-based practice in high-risk cardiac surgical care.

## Supplementary Material

1

Supplementary material associated with this article can be found in the online version at doi:10.1053/j.jvca.2025.08.013.

## Figures and Tables

**Table 1:** Characteristics of patients undergoing cardiac surgery with vs without pulmonary arterial catheterization

Characteristic	No PAC	PAC	p value*
Age, y, mean ± SD [95% CI]	60.55 ± 14.74 [0.41–60.69]	62.54 ± 12.83 [62.46–62.62]	<0.0001
BMI, mean ± SD [95% CI]	28.96 ± 5.88 [28.90–29.01]	29.02 ± 6.00 [28.98–29.06]	0.0634
Weight, kg, mean ± SD [95% CI]	86.07 ± 20.42 [85.88–86.27]	86.66 ± 20.65 [86.54–86.79]	<0.0001
Sex, n (%)			
Female	13,421 (33.0)	32,292 (30.9)	<0.0001
Male	27,296 (67.0)	72,334 (69.1)	
Race/ethnicity, n (%)			
American Indian or Alaska Native	222 (<1)	437 (<1)	<0.0001
Asian or Pacific Islander	1,360 (3.3)	3,418 (3.3)	
Multiracial	196 (<1)	985 (<1)	
Black (not Hispanic)	3,350 (8.2)	9,863 (9.4)	
Hispanic black	36 (<1)	129 (<1)	
White (Hispanic)	636 (1.6)	1,272 (1.2)	
Middle Eastern	12 (<1)	35 (<1)	
Unknown	3,926 (9.6)	16,344 (15.6)	
White (not Hispanic)	30,979 (76.1)	72,143 (69.0)	
ASA score, n (%)			
1	29 (<1)	20 (<1)	<0.0001
2	505 (1.2)	565 (<1)	
3	10,605 (26.0)	23,499 (22.5)	
4	28,857 (70.9)	78,137 (74.7)	
5	721 (1.8)	2,405 (2.3)	
Annual surgical volume, mean ± SD [95% CI]	962.37 ± 593.26 [956.61–968.13]	1009.95 ± 552.95 [1006.60–1013.30]	<0.0001
Weekday, n (%)	39,374 (96.7)	99,584 (95.2)	<0.0001
Weekend, n (%)	1,343 (3.3)	5,042 (4.8)	
CRNA started case, n (%)	4,936 (12.1)	16,314 (15.6)	<0.0001
Resident started case, n (%)	21,462 (52.7)	67,106 (64.1)	<0.0001
Emergent case, n (%)	2,288 (5.6)	9,039 (8.6)	<0.0001
Albumin, g/dL, mean ± SD [95% CI]	4.09 ± 1.70 [4.08–4.11]	4.13 ± 1.93 [4.12–4.14]	0.0009
BUN, mg/dL, mean ± SD [95% CI]	19.00 ± 9.90 [18.91–19.10]	20.96 ± 11.19 [20.89–21.03]	<0.0001
K^+^, mEq/L, mean ± SD [95% CI]	4.20 ± 0.44 [4.19–4.20]	4.21 ± 0.42 [4.21–4.22]	<0.0001
Hgb A1c, %, mean ± SD [95% CI]	6.18 ± 1.21 [6.17–6.19]	6.18 ± 1.15 [6.17–6.19]	0.9984
Glucose, md/dL, mean ± SD [95% CI]	116.47 ± 38.74 [116.09–116.84]	118.06 ± 38.87 [117.82–118.29]	<0.0001
Hgb, g/dL, mean ± SD [95% CI]	13.14 ± 2.05 [13.12–13.16]	12.81 ± 2.11 [12.80–12.82]	<0.0001
INR, mean ± SD [95% CI]	1.11 ± 0.34 [1.10–1.11]	1.14 ± 0.27 [1.14–1.14])	<0.0001
Platelet count, per/μL, mean ± SD [95% CI]	222.89 ± 74.71 [222.16–223.61]	216.51 ± 74.05 [216.06–216.96]	<0.0001
Arrhythmia, n (%)	24,098 (59.2)	71,083 (67.9)	<0.0001
Chronic pulmonary disease, n (%)	7,888 (19.4)	24,149 (23.1)	<0.0001
Congestive heart failure, n (%)	15,438 (37.9)	54,576 (52.2)	<0.0001
Peripheral vascular disease, n (%)	10,744 (26.4)	41,040 (39.2)	<0.0001
Pulmonary circulation disorder, n (%)	4,406 (10.8)	19,726 (18.9)	<0.0001
Valvular disease, n (%)	20,878 (51.3)	65,897 (63.0)	<0.0001
Cerebrovascular disease, n (%)	4,996 (12.3)	16,864 (16.1)	<0.0001
Coronary arterial disease, n (%)	24,714 (60.7)	63,566 (60.8)	0.8376
Isolated CABG, n (%)	19,411 (47.7)	44,384 (42.4)	<0.0001
CABG, n (%)	20,328 (49.9)	47,901 (45.8)	<0.0001
MV repair, n (%)	4,694 (11.5)	12,227 (11.7)	0.3989
MV replacement, n (%)	3,236 (7.9)	13,727 (13.1)	<0.0001
Tricuspid repair/replacement, n (%)	2,407 (5.9)	5,961 (5.7)	0.1156
Pulmonic repair/replacement, n (%)	955 (2.3)	411 (<1)	<0.0001
AV replacement, n (%)	7,438 (18.3)	26,370 (25.2)	<0.0001
AVSS or AV repair, n (%)	4,578 (11.2)	13,607 (13.0)	<0.0001
Proximal aortic, n (%)	5,800 (14.2)	17,688 (16.9)	<0.0001
Descending thoracic aortic, n (%)	291 (<1)	914 (<1)	0.0027
Lung transplant, n (%)	681 (1.7)	4,528 (4.3)	<0.0001
Heart transplant, n (%)	309 (<1)	4,480 (4.3)	<0.0001
Redo sternotomy, n (%)	1,820 (4.5)	6,019 (5.8)	<0.0001

Abbreviations: ASA, American Society of Anesthesiology; AV, aortic valve; AVSS, aortic valve−sparing surgery; BMI, body mass index; BUN, blood urea nitrogen; CABG, coronary artery bypass graft; CI, confidence interval; CRNA, Certified Registered Nurse Anesthetist; Hgb, hemoglobin; INR, International Normalized Ratio; MV, mitral valve; PAC, pulmonary arterial catheter; SD, standard deviation.

* Statistical significance does not necessarily imply a clinically meaningful difference.

**Table 2:** Factors associated with pulmonary arterial catheterization

Characteristic (fixed effects)	Anesthesiologist plus patient characteristics, OR (95% CI)	Anesthesiologist within hospital plus patient characteristics, OR (95% CI)
Year		
2016 (reference)	—	—
2017	0.79 (0.69–0.90)	0.80 (0.71–0.92)
2018	0.64 (0.56–0.74)	0.69 (0.60–0.78)
2019	0.59 (0.51–0.69)	0.65 (0.56–0.75)
2020	0.69 (0.60–0.81)	0.69 (0.60–0.80)
2021	0.62 (0.53–0.72)	0.61 (0.53–0.70)
2022	0.52 (0.45–0.61)	0.50 (0.43–0.58)
Annual surgical volume*	1.23 (1.15–1.32)	1.24 (1.15–1.33)
Age (years)	1.01 (1.01–1.01)	1.01 (1.01–1.02)
Sex		
Female (reference)	—	—
Male	1.23 (1.15–1.32)	1.24 (1.15–1.33)
Race/ethnicity		
American Indian (reference)	—	—
Asian or Pacific Islander	1.02 (0.64–1.60)	0.98 (0.62–1.53)
Biracial/multiracial	0.98 (0.55–1.77)	0.80 (0.45–1.43)
Black not Hispanic	1.19 (0.77–1.84)	1.17 (0.76–1.81)
Black Hispanic	1.16 (0.41–3.30)	1.03 (0.36–2.96)
White Hispanic	0.94 (0.57–1.54)	0.93 (0.56–1.53)
Middle Eastern	0.99 (0.13–7.19)	1.60 (0.19–13.30)
Unknown	1.07 (0.69–1.65)	1.00 (0.65–1.54)
White not Hispanic	0.93 (0.61–1.43)	0.92 (0.60–1.40)
BMI	1.01 (1.00–1.02)	1.01 (1.01–1.02)
ASA status		
1 (reference)	—	—
2	1.96 (0.38–10.10)	1.77 (0.38–8.21)
3	3.32 (0.66–16.61)	2.86 (0.64–12.87)
4	5.19 (1.04–25.98)	4.56 (1.02–20.44)
5	4.79 (0.94–24.35)	4.01 (0.88–18.40)
Weekend case	1.20 (1.00–1.44)	1.21 (1.01–1.45)
CRNA started case	0.99 (0.86–1.13)	0.76 (0.67–0.87)
Resident started case	1.06 (0.98–1.16)	0.94 (0.86–1.02)
Emergency case	1.05 (0.90–1.23)	1.03 (0.88–1.20)
Cardiac arrhythmia	1.07 (1.00–1.14)	1.06 (0.99–1.14)
CPD	1.09 (1.01–1.18)	1.11 (1.03–1.20)
CHF	1.79 (1.67–1.92)	1.81 (1.69–1.94)
PVD	1.68 (1.56–1.82)	1.62 (1.50–1.76)
Valvular pathology	1.21 (1.12–1.31)	1.18 (1.09–1.27)
CVD	1.16 (1.06–1.28)	1.15 (1.05–1.26)
CAD	1.01 (0.93–1.10)	1.02 (0.93–1.11)
BUN (mg/dL)	1.01 (1.01–1.01)	1.01 (1.00–1.01)
Potassium (mEq/L)	0.92 (0.85–0.99)	0.92 (0.86–1.00)
Hgb A1c (%)	1.05 (1.02–1.08)	1.06 (1.03–1.09)
INR	1.08 (0.95–1.21)	1.10 (0.96–1.26)
Hgb (g/dL)	0.99 (0.97–1.00)	0.99 (0.97–1.00)
Isolated CABG	1.31 (1.19–1.43)	1.37 (1.25–1.50)
MV repair	1.42 (1.27–1.58)	1.46 (1.31–1.63)
MV replacement	2.32 (2.07–2.59)	2.38 (2.13–2.67)
AV replacement	1.77 (1.60–1.96)	1.81 (1.64–2.00)
Tricuspid	0.74 (0.65–0.85)	0.77 (0.67–0.88)
AVSS or AV repair	1.01 (0.89–1.13)	1.00 (0.89–1.13)
Proximal aortic surgery	1.85 (1.67–2.05)	2.14 (1.93–2.39)
Lung transplant	9.58 (7.58–12.15)	8.94 (7.12–11.25)
Heart transplant	9.70 (7.12–13.30)	10.72 (7.77–14.81)
Redo sternotomy	1.48 (1.28–1.71)	1.53 (1.32–1.76)
Anesthesiologist^†^	7.70 (6.82–8.76)	—
Anesthesiologist within hospital^‡^	—	1.70 (1.61–1.81)
Hospital^§^	—	15.00 (8.98–28.32)

Abbreviations: ASA, American Society of Anesthesiology; AV, aortic valve; AVSS, aortic valve−sparing surgery; BMI, body mass index; BUN, blood urea nitrogen; CABG, coronary artery bypass graft; CAD, coronary artery disease; CHF, congestive heart failure; CPD, chronic pulmonary disease; CRNA, Certified Registered Nurse Anesthetist; CVD, cardiovascular disease; Hgb, hemoglobin; INR, International Normalized Ratio; MOR, median odds ratio; MV, mitral valve; OR, odds ratio; PAC, pulmonary arterial catheter; PVD, peripheral vascular disease.

* Surgical volume was divided by 10 to produce more meaningful ORs.

† In the 2-level model, the influence of the anesthesiologist was summarized using the MOR, which is the odds of PAC receipt between 2 otherwise identical patients cared for by 2 randomly selected anesthesiologists across all hospitals.

‡ This MOR indicates the odds of PAC receipt among 2 otherwise identical patients being cared for by 2 randomly selected anesthesiologists practicing in the same hospital.

§ This MOR indicates the odds of PAC receipt among 2 otherwise identical patients undergoing surgery at 2 randomly selected hospitals and with different anesthesiologists.
