# Supplementary material for: Practice Pattern Variability in the Use of Pulmonary Arterial Catheters in Cardiac Surgery
Source: J Cardiothorac Vasc Anesth. Author manuscript; Available in PMC 2026 Apr 7. (PMC13056024; doi:10.1053/j.jvca.2025.08.013)
Supplement: 1 [file NIHMS2162374-supplement-1.docx]

**eSupplement**

**Section 1: National Multicenter Perioperative Outcomes Group (MPOG) Data Information**

**1.1: MPOG Research Standard at the time of data query**

Valid anesthesia start/end times

Valid patient in-room time

Common Procedural Technology (CPT) codes available

Documented age, sex, and American Society of Anesthesiologists Physical Status (ASA-PS) classification

At least one blood pressure measurement

At least one intraoperative medication

At least one International Classification of Diseases (ICD-9/10) discharge diagnosis code

At least one serum creatinine or hematocrit obtained within 365 days before or after surgery

**1.2: Link to MPOG Adult Cardiac Phenotype**

<https://phenotypes.mpog.org/Procedure%20Type:%20Cardiac%20(Adult)>

**Section 2: GitHub Repository for PAC Variability**

<https://github.com/emily-jane-mackay/PAC_Variability>
